# The Effect of the Microstructure Formed in the Forging–Healing Process on the Mechanical Properties of Heavy Forgings

**DOI:** 10.3390/ma16155205

**Published:** 2023-07-25

**Authors:** Qianjun Liu, Yao Qiu, Ruishan Xin, Jianbin Luo, Qingxian Ma

**Affiliations:** 1 Key Laboratory for Advanced Materials Processing Technology of Ministry of Education, Department of Mechanical Engineering, Tsinghua University, Beijing 100084, China; 2 Ansteel Beijing Research Institute Co., Ltd., Beijing 102211, China

**Keywords:** plastic deformation, heavy forgings, mechanical properties, defect healing

## Abstract

The forging–healing of the internal porosity defects affects the tensile, impact and fatigue properties of heavy forgings. In the present work, the effect of deformation process on the microstructure in the joint area as well as the tensile strength, impact toughness and fatigue strength was studied experimentally. It is shown that the tensile strength is restored once the porosity defects were healed, and the impact toughness is recovered when the flat grain band is eliminated. The fatigue strength can be restored if a uniform grain structure can be achieved in both the joint area and the matrix, whereafter precipitate become the key factor affecting the fatigue strength. A complete healing of the porosity defects, a uniform grain structure in the joint area and the matrix, and a fully controlled precipitate are essential to guarantee the mechanical properties and in-service performance of the heavy forgings.

## 1. Introduction

As load-bearing components, forgings are widely used in energy equipment manufacturing, shipbuilding and other important fields. The quality and performance of the forgings determine the reliability of the equipment. The mechanical properties of the forgings depend on their chemical composition and structures. Therefore, it is very important to study the influence of plastic deformation processes on the structures as well as their corresponding effects on the tensile, impact and fatigue properties, which is essential for providing guidelines to formulate appropriate forging processes to guarantee the comprehensive mechanical properties and service life of the forgings [1,2,3,4,5].

Nowadays, ingots produced using double vacuum smelting process are used to produce heavy forgings, where inclusions are normally well controlled. However, due to the solidification shrinkage or the thermal expansion of the materials, cavity or porosity defects exist in the ingots inevitably, and with the increase in the size of the ingots, it becomes more difficult to control the porosity defects. The forging–healing of the porosity defects has remarkable influences on the tensile, impact and fatigue properties of the forgings, especially for super heavy forgings. Accordingly, a precise control of the healing quality becomes the key factor ensuring the internal quality, load bearing capacity and service performance of the forgings. A508.3 steel is generally used to manufacture pressure vessel forgings for nuclear reactors. The comprehensive mechanical properties of forgings, such as tensile strength, impact toughness, fatigue strength, etc., play an important role in reactor safety, which has attracted great attention.

It is the most feasible and efficient method of hot plastic deformation for mechanical properties required in the actual production of heavy forgings. Han et al. [6] found that the mechanism of recovery process was the diffusion and migration of metal atoms by using the experimental modelling in 20MnMo steel specimens with inner crack at high temperature. Yu et al. [7] pointed out that the recovery degree of the internal crack increased when increasing heating temperature, holding time and reduction ratio and decreased with the increase in deformation passes and strain rate, by studying the influence of different process parameters on crack healing in low-carbon steel specimens under hot deformation. Wei et al. [8] studied the morphology evolution of steel specimens with internal cracks by quasi in situ observation and pointed out that the crack healing at crack tips could be achieved after healing at 1100 °C for 120 min. Zhang et al. [9] studied the microstructure and hardness with artificially created internal cracks and found that, by using hot compression and heat treatment, the ferrite grains assembled along the boundary of the crack, and sub-grains had higher hardness in the healing ranges than the matrix. Recently, the crack healing mechanism in metallic materials were also studied by a molecular dynamics model [10,11]. Some investigations on mechanical properties recovered after crack healing are reported recently. In our previous study [12,13,14], we found the relationship between tensile strength, impact toughness and changes in grain structure at the interface of porous defects. Xin et al. [12] discovered the phenomena that when internal microcracks disappeared completely under heat treatment healing technique, the tensile properties of the crack healing zone recovered completely, but the impact toughness could only partially be restored. Qiu and Liu et al. [13,14] pointed out that dynamic load performance recovery such as impact toughness not only requires the elimination of porosity defects but also requires the grain structure of the repair interface to be basically the same as the matrix structure. By using the experimental modeling and simulation, the influence of process parameters such as plastic deformation, insulation temperature, repair time and deformation rate on impact toughness was obtained.

However, the study about the recovery of mechanical properties under dynamic load, which is important for actual service condition of heavy forgings, is still not enough, and it is essential to carry out further research on crack healing methods to improve mechanical properties recovery. The effects of deformation modes and quenching and tempering (Q&T) process on internal crack healing in SA 508-3 steel were studied, which laid the foundation to further study optimum internal crack healing strategy.

For a certain steel grade with a particular chemical composition, the forging process parameters including deformation amount, temperature and deformation rate determine the microstructure of the forgings. It is of significance to investigate the relationship among the process parameters, microstructures and the mechanical properties under static and dynamic loads, i.e., tensile strength, impact toughness and fatigue strength, which is a knowledge base for exploring approaches to increase the load bearing performance and improve the in-service safety of the forgings.

In the present work, the forging–healing of the internal porosity defects was studied as well as the effect of the deformation process parameters and microstructures on the tensile strength, impact toughness and fatigue strength of the forgings.

## 2. Material and Experimental Method

**Figure 1:**
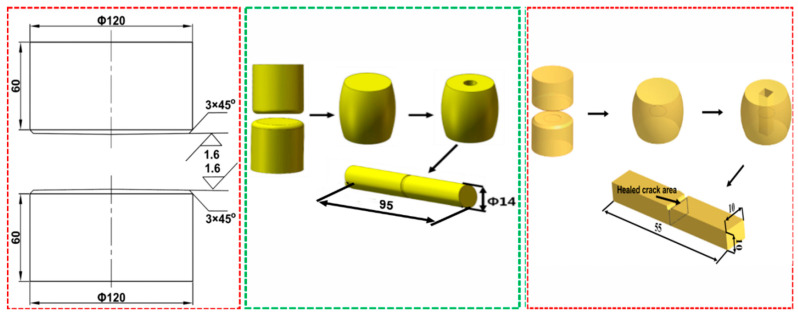
The geometry of the cylindrical pieces and the sampling illustration for property test.

An experimental method called butt-joint and high-temperature compression method was employed to simulate the forging–healing process, by which the effect of the process parameters and microstructure on the tensile strength, impact toughness and fatigue strength was studied. SA508.3 forged steel was chosen as the experimental material and cylindrical pieces with a diameter of 120 mm and a height of 60 mm were cut from the forged material. The chemical composition (%, mass fraction) of the material cut from the SA508.3 steel forging is C 0.19, Si 0.22, Mn 1.4, P 0.006, S 0.006, Cr 0.12, Mo 0.53, Ni 0.53 and the balance is Fe. The high-temperature forging experiment was conducted by using two cylindrical pieces welded at the edges. The geometry of the cylindrical piece and the forging experiment process are shown in Figure 1.

In order to evaluate the forging–healing effectiveness, experiments for the purpose of comparison were designed and conducted, which means that for any experiment, a counterpart forging experiment was carried out under the same deformation condition. We may consider the joint interface between the two cylindric pieces to be an artificial crack, and in the counterpart experiment, the work piece is a single bulk of the material without any artificial crack. In addition, a term ‘percentage of recovery’ was defined as the ratio of the property value measured in the experiment to that measured in the counterpart experiment.

**Figure 2:**
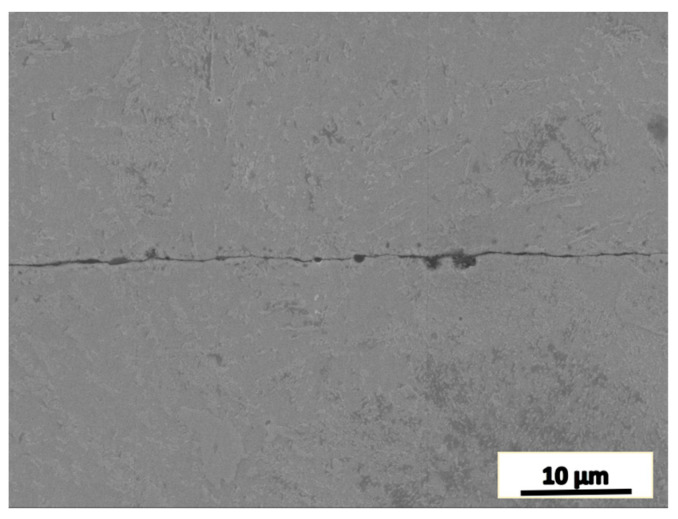
SEM micrograph of the joint zone before the plastic deformation.

Figure 2 shows the morphology of the original joint zone in the cylindrical pieces. The width of the original internal joint area was about 0.5 µm in the middle part. 

The cylindric work pieces were forged under designed processes, held at a certain temperature and cooled to room temperature. Specimens for mechanical property test were taken from the forged work piece by using wire cutting along the axial direction of the piece.

**Figure 3:**
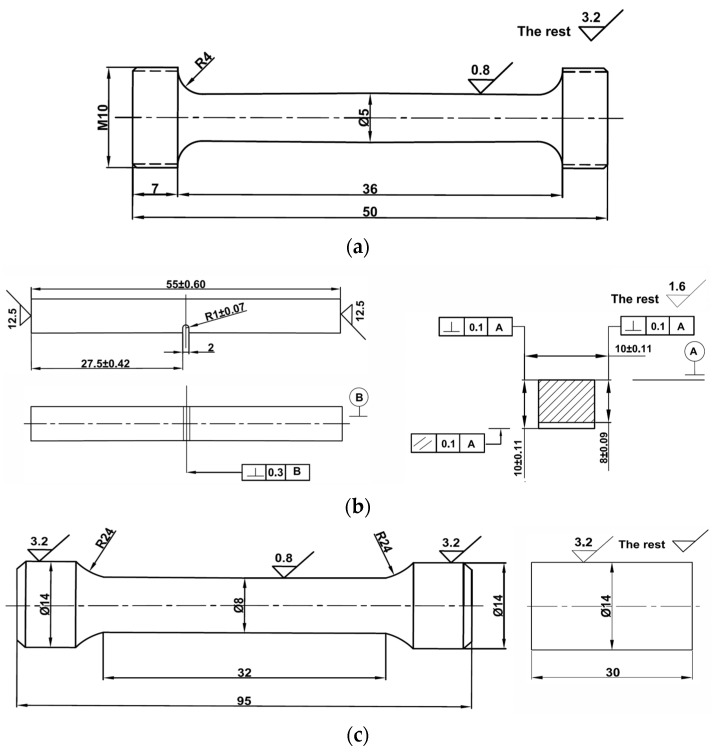
The geometry and dimensions of the mechanical property test specimens. (**a**) The tensile test specimen, (**b**) The impact test specimen, (**c**) The low cycle fatigue test specimen.

The geometry and dimensions of the tensile test specimens are shown in Figure 3a according to the China National Standard GB/T228.1 ‘Tensile Test of Metallic Materials Part 1: Room Temperature Test Method’ [15]. The tensile tests at room temperature were carried out on a 30-ton universal material test machine.

According to the China National Standard GB/T229 ‘Metallic Materials Charpy Impact Test Method’ [16], the size of the impact test specimen is 10 mm × 10 mm × 55 mm, and a U-shaped notch was created at the middle position of the length of the specimen, where the joint interface or healed crack is located, as shown in Figure 3b. The impact test was carried out on a 300 J pendulum impact test machine.

According to the China National Standard GB/T 15248-2008 ‘Axial Constant Amplitude Low Cycle Fatigue Test Method for Metallic Materials’ [17], the specimens for fatigue test with geometry and dimensions shown in Figure 3c were prepared. The fatigue test was carried out using an INSTRON low cycle fatigue test machine. In the test, a constant strain triangular wave load was employed, where the constant strain amplitude is 0.006, the load frequency is 0.83 Hz, the strain rate is 0.02 s^−1^, and the load ratio Rs is −1. The specimen was broken when the load dropped to 25% of the stable load.

## 3. The Effect of Deformation Process Parameters on the Tensile Strength

### 3.1. Effect of Tensile Strength Recovery under Different Deformation Conditions

**Figure 4:**
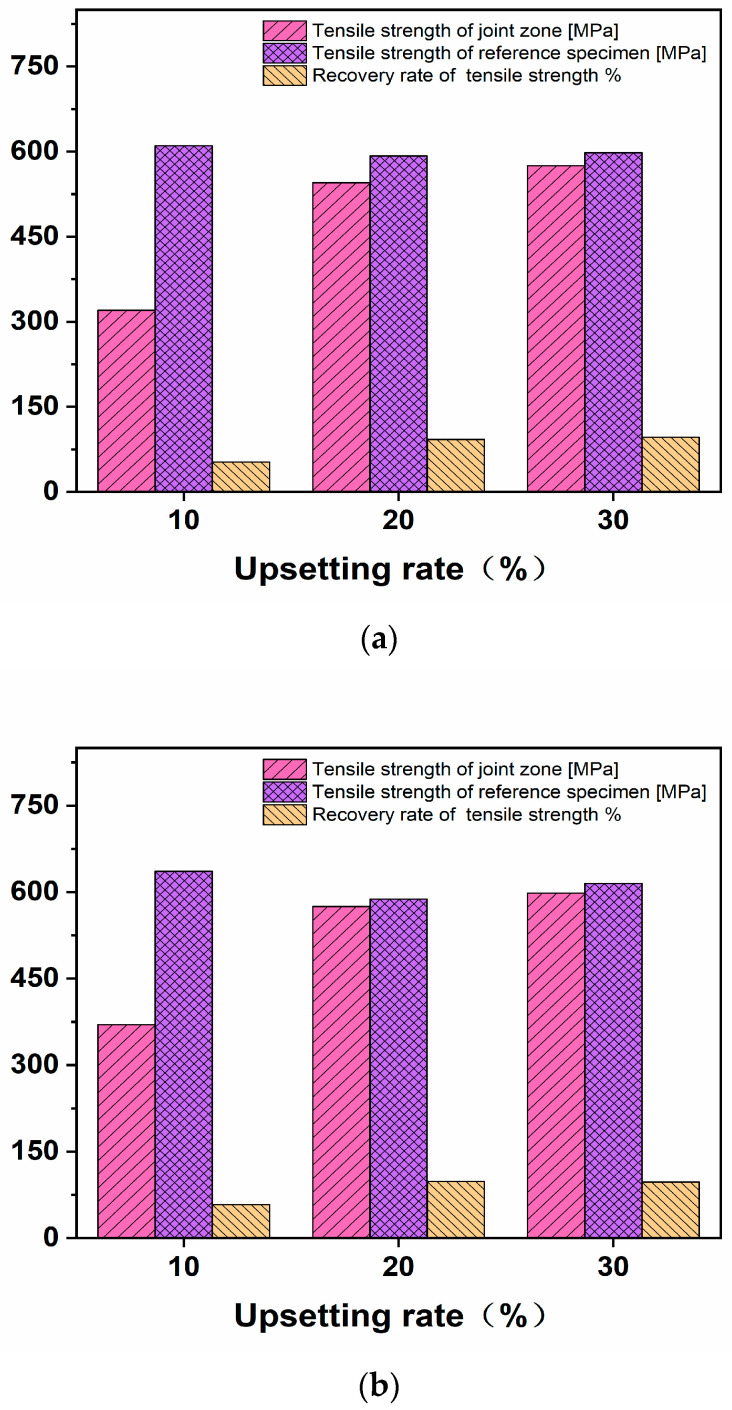

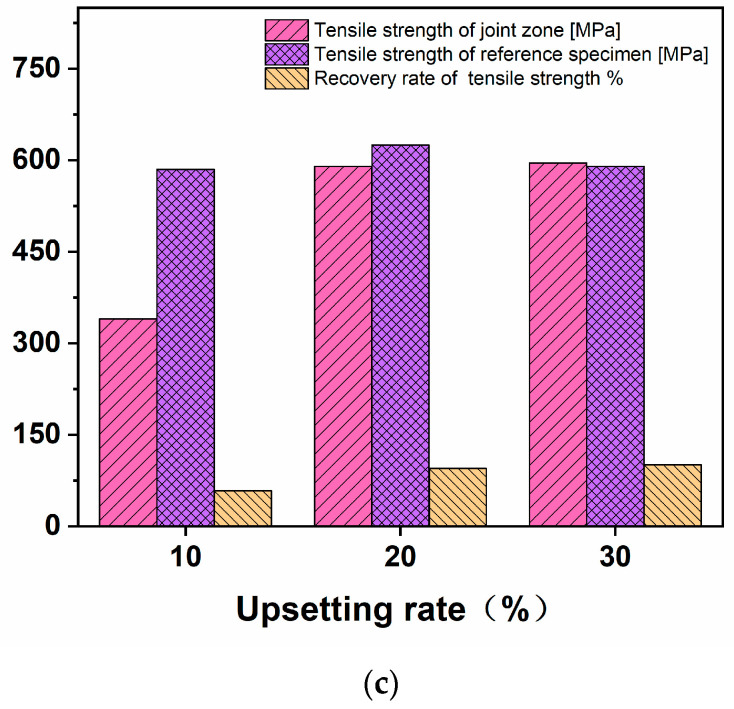
Percentage recoveries of room-temperature tensile strength under different deformation modes. (**a**) 950 °C, (**b**) 1050 °C, (**c**) 1150 °C.

The specimens were heated to 950, 1050 and 1150 °C for upsetting deformation, and the upsetting reduction rates were 10%, 20% and 30%, respectively. After the forging is completed, keep it at the deformation temperature for 60 min, and then air cool it to room temperature. According to the method and location in Figure 1, the specimen is sectioned for tensile deformation, and the tensile strength is obtained as shown in Table 1. The percentage recoveries of room-temperature tensile strength under different deformation modes are shown in Figure 4.

The tensile strength of the specimen decreases with the increase in deformation temperature. When the deformation temperature is the same and the upsetting reduction rate is 20%, the tensile strength of the material can basically recover to the initial structure state. Under the experimental temperature conditions, the effect of temperature on the recovery of tensile strength is not significant.

### 3.2. The Effect of Deformation Amount on the Tensile Property

**Figure 5:**
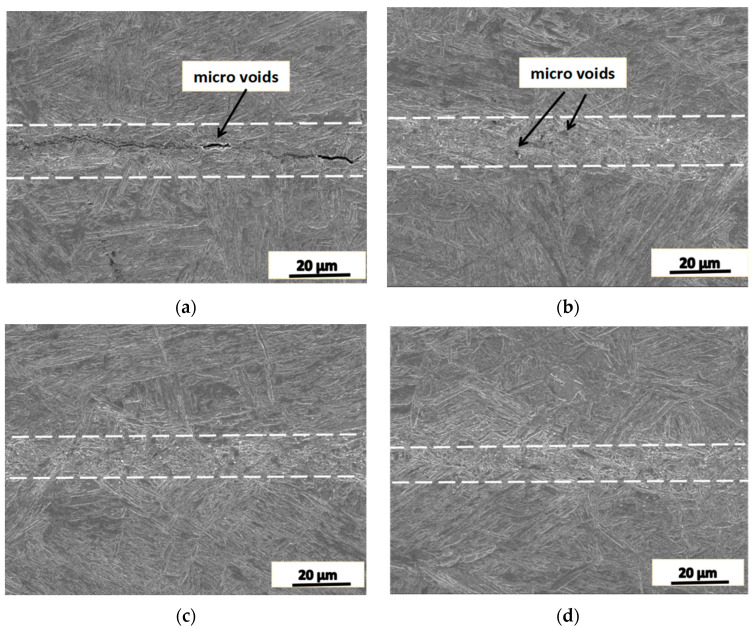
The microstructure of the joint area of SA508.3 steel after forging–healing with different reduction rate, where the temperature is 1050 °C and the deformation rate is 0.1 s^−1^. (**a**) 5%, (**b**) 10%, (**c**) 20%, (**d**) 30%.

The cylindrical piece was heated to 1050 °C and held at this temperature for 60 min, followed by upsetting deformation and natural cooling to room temperature. Figure 5 shows the SEM image of the microstructure of the joint area.

As shown in Figure 5a,b, when the reduction rate of the cylindrical piece is less than 10%, the gap of the joint interface reduced gradually, and the microstructure of the joint area is composed of fine grains, and the width of the fine grain structure band is about 10 μm. However, micropores remained in the fine grain band (see Figure 5b). When the reduction rate reaches 20% (Figure 5c), the micropores in the joint area disappeared, and the microstructure in this area became finer. When the reduction rate reaches 30%, the fine grain structure almost disappeared, as shown in Figure 5d, where no apparent difference between the joint area and the matrix was observed. This means that the high reduction rate makes the joint interface fully healed. Tensile test showed that the tensile strength is almost the same as that of the counterpart experiment where the forged material does not have any artificial crack, i.e., the tensile strength was restored after the porosity defect was healed by forging.

The comparison of the structure state shows that the tensile strength basically recovers after the elimination of internal porosity defects. Temperature and plastic deformation are important factors that affect the forging of internal porous defects to improve the tensile strength. The effect is mainly reflected by the existence of porous defects.

### 3.3. The Effect of the Deformation Rate on Crack Healing

**Figure 6:**
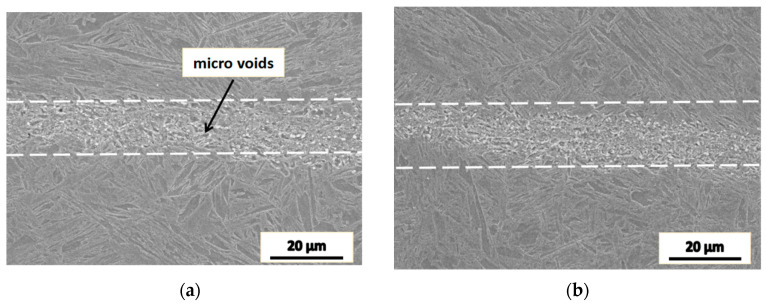
The microstructure of the joint area with different strain rate, the reduction rate 30% and deformation temperature 1050 °C. (**a**) 1 s^−1^ and (**b**) 0.01 s^−1^.

The cylindrical piece was compressed by the reduction rate of 30% at 1050 °C with different strain rates, and the microstructure of the joint area is shown in Figure 6.

When the strain rate is 1 s^−1^, the joint interface area is uneven, and there are many small pores in it, as shown in Figure 6a. This was mainly caused by the high strain rate that made the work piece deform rapidly. The accumulated strain and energy cannot be released through recrystallization in the joint area. When the applied strain rate is 0.01 s^−1^, the unevenness in the joint area was significantly reduced, as shown in Figure 6b, and there are no micropores in the area while the microstructure is still different from the matrix. With the decrease in the applied strain rate, the width of the fine grain structure band increased, the pores in the band decreased even disappeared, and the unevenness of the joint area decreased as well.

Under the condition of the same reduction, a decrease in the strain rate means an increase in the deformation time, which allows a longer time for recrystallization nucleation and growth in the joint area. The recrystallized grains fill the cracks and pores, thus reducing the unevenness in the joint area.

It was found that the thermoplastic deformation resulted in the healing of the joint interface; however, microstructure differences still exist between the joint area and the matrix. Therefore, for the steel SA508.3, a treatment of holding at 1050 °C for a period of time is necessary to promote homogenization and reduce the microstructure difference between the joint area and the matrix, though the tensile strength does not have a noticeable change in this process.

Based on the above results shown in Figure 5 and Figure 6, it was found that the forging–healing and the recrystallization achieved a microstructure bonding in the joint area, the tensile strength of the joint restored to strength level of the matrix of the material, and a slight change in the microstructure does not cause obvious change in the tensile strength.

**Figure 7:**
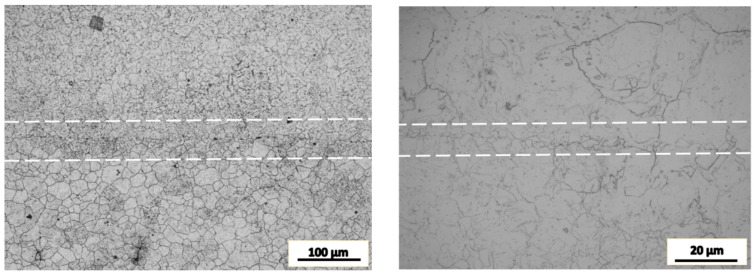
Critical state of tensile strength of typical porous structure.

At this time, typical defect repair tissues were cut for impact test, as shown in Figure 7. The corresponding impact toughness recovery value is about 60% of the comparative value. The grain boundary distribution of metal structure with “I” shape is an important cause of impact damage.

## 4. The Effect of the Microstructure in Joint Area on the Impact Toughness

The cylindrical pieces were heated to 950, 1050 and 1150 °C, respectively, followed by holding at the temperatures for 60 min, and then the pieces were treated with the following processes: upsetting, upsetting–drawing–upsetting and upsetting–drawing–upsetting–drawing, respectively. The reduction rate of the upsetting is 20%, and the reduction sequence in each drawing cycle is 0°→180°→90°→270°. One drawing operation is composed of four reduction cycles, and a full anvil reduction was applied with reduction rate of 20% for each pressing. After the forging process, the pieces were holding at the deformation temperature for 60 min, and then were cooled naturally to room temperature.

### 4.1. Impact Toughness Recovery under Different Deformation Modes

**Figure 8:**
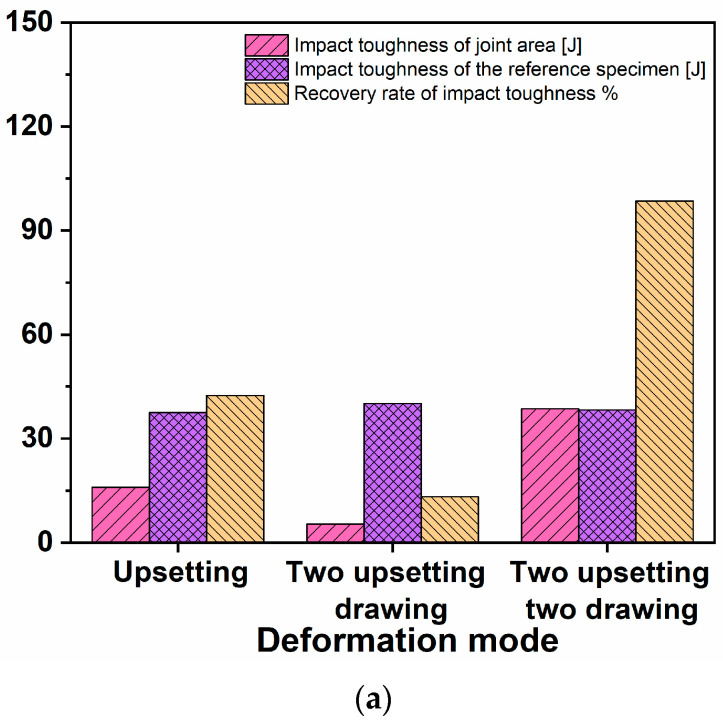

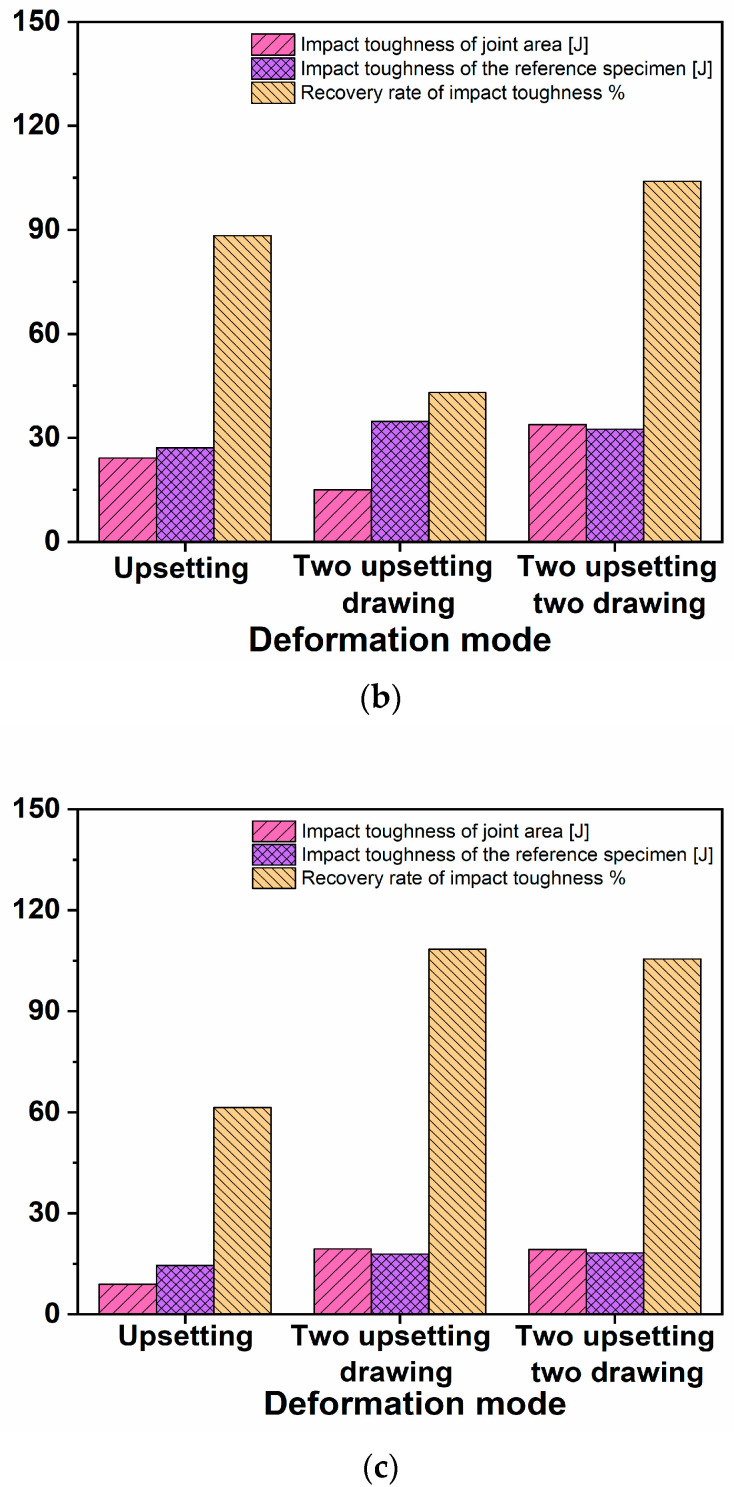
Percentage recoveries of room-temperature impact properties under different deformation modes. (**a**) 950 °C, (**b**) 1050 °C, (**c**) 1150 °C.

According to the test, the recovery of the impact properties of the section bonding zone under different deformation modes is shown in Table 2. The percentage recoveries of room-temperature impact properties under different deformation modes are shown in Figure 8.

When the deformation mode is the same, the impact property of the control specimen decreases with the increase in deformation temperature. When the deformation temperature is the same, the impact absorption energy of the comparison specimen after two upsetting and one drawing deformation is significantly increased compared with that of the first upsetting, and the impact absorption energy of the simulated defect specimen returns to the initial state at 1150 °C. The impact absorbing energy of the specimen after two upsetting and two drawing deformation is basically the same as the impact performance of the comparison specimen, which indicates that the impact performance of the defect is basically recovered.

It can be seen from this that temperature and plastic deformation are important factors that affect the forging of internal porosity defects to improve the impact performance. Obviously, it is more difficult to repair the dynamic mechanical properties such as impact than the static mechanical properties such as tension and compression.

### 4.2. The Microstructure of the Joint Area with Impact Toughness Recovered

**Figure 9:**
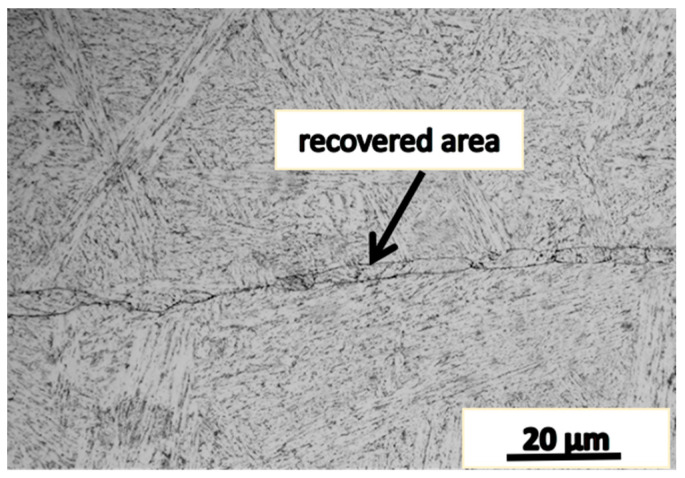
The microstructure near the fracture area of the specimen with the impact toughness recovered to that of the matrix material.

Figure 9 shows the microstructure near the fracture area of the specimen of which the impact absorption energy was approximately recovered to that of the matrix material.

It can be seen from Figure 9 that the joint interface of the specimen features a discontinuous wave morphology, which increases the difficulty for crack propagation and thus leads to the impact absorption energy recovery. Inclusions containing Al and Si were found in the joint area, which is considered a factor having a negative effect on the recrystallization and the recovery of the impact absorption energy.

By comparing with the reported work [6], we may understand that when the deformation mode is upsetting the microstructure of the joint area is mainly a relatively flat grain band. When the deformation mode is upsetting–drawing–upsetting, the grains in the band have particular orientation and their size has a large difference with the matrix, which is also called mixed crystals, and with the increase in the process temperature, the impact absorption energy and the recovery rate of impact energy are increased. In both these processes, the microstructure are uneven while the upsetting–drawing–upsetting process has a better effectiveness.

When the deformation mode is the same, the impact absorption energy of the specimen decreases with the increase in the deformation temperature. When the deformation temperature is the same, the impact absorption energy of the specimen processed by upsetting–drawing–upsetting was increased significantly as compared with that by only one upsetting. When the deformation temperature is 1150 °C, the impact absorption energy of the specimen was fully restored. When the upsetting–drawing–upsetting–drawing process was applied, the impact absorption energy of the specimen was also fully restored, and it was noticed that the microstructure of the joint area is almost the same as that in the counterpart experiments.

The deformation temperature, amount and time are the important factors affecting the healing of internal porosity defects and changing the impact property, and the microstructure of the joint area, a bridge of the process and property, accounts for the difference in the recovery mechanism in the tensile and impact properties.

### 4.3. The Requirements for Deformation Amount

**Figure 10:**
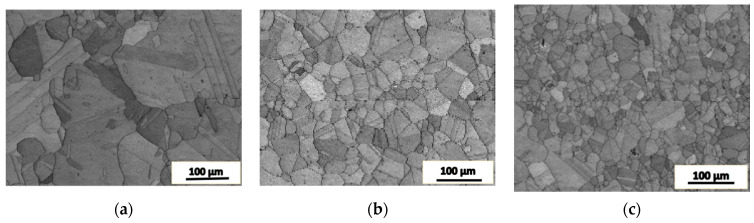
The microstructure of SA508.3 steel deformed at 1050 °C under different strain conditions. (**a**) 0.1, (**b**) 0.3, (**c**) 0.7.

Figure 10 shows the microstructure of SA508.3 steel after upsetting deformation at 1050 °C where the deformation strains are 0.1, 0.3 and 0.7 calculated by the finite element method, respectively. In the early stage of deformation, the grain boundaries were bended, and recrystallization appears at some triple grain boundaries, as shown in Figure 10a. With the increase in the strain, a large number of dynamic recrystallized nuclei appeared along the original grain boundaries and began to grow into the grains (see Figure 10b). When the deformation strain reached 0.7, the original structure was completely replaced by newly generated dynamic recrystallized grains, as shown in Figure 10c. The evolution of the microstructure can be divided into the following three stages.

1. In the early stage of the deformation, the strain was small and the grain boundaries began to bend, which created conditions of energy and structure for the nucleation of dynamic recrystallization.
2. With the increase in the strain, a large quantity of sub-grains were formed near the original grain boundaries, and the sub-grains formed the nuclei of dynamic recrystallization.
3. In the consequent deformation process, the recrystallized nuclei grew up through the migration of large angle grain boundaries and finally replaced the original grain structure.

It implies that sufficient plastic deformation and time are required to achieve a similar grain size in both the matrix and the joint area. In other words, the recovery of the impact property requires sufficient plastic deformation and holding time for homogenization.

**Figure 11:**
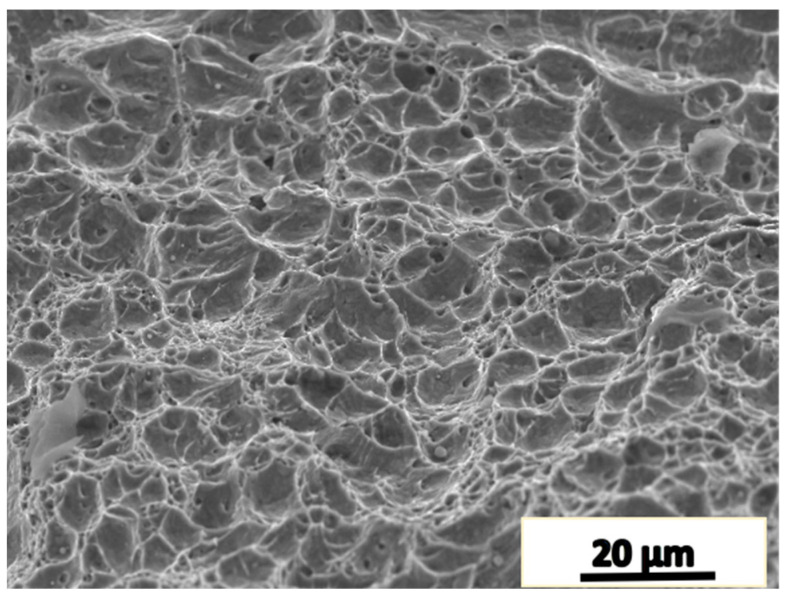
Dimples appear on the impact fracture surface.

The microstructure of the joint surface of the two upsetting and two drawing forgings is shown in Figure 9. After the microstructure is uniform, the impact toughness is restored. Figure 11 shows the fracture of the specimen with the impact property restored. The morphology of the fracture is characterized by dimples and cleavage facets, and the fraction of dimples is higher, thus resulting in a high impact absorption energy.

## 5. The Effect of Defect Healing on the Fatigue Property

The cylindrical pieces were heated to 950, 1050 and 1150 °C, respectively, followed by holding at the temperatures for 60 min, and then the pieces were treated with the processes of upsetting–drawing–upsetting–drawing. The reduction rate of the upsetting is 20%, and the reduction sequence in each drawing cycle is 0°→180°→90°→270°. One drawing operation is composed of four reduction cycles, and a full anvil reduction was applied with reduction rate of 20% for each pressing. After the forging process, the pieces were holding at the deformation temperature for 1 h, and then were cooled naturally to the room temperature. Counterpart experiments were conducted for evaluating the recovery percentage of the fatigue property.

For the pieces deformed at 950 °C and 1150 °C, the fatigue cycles show a remarkable difference under the test condition with the strain amplitude of 0.0064. The number of fatigue cycles of the pieces deformed at 950 °C is 3566, while that of the pieces deformed at 1150 °C is 26,522. It was noticed that the fracture does not always appear in the joint area, which indicates that the fatigue property of the joint area has no difference from that of the matrix. Inclusions and carbides were found in the fracture and there is an apparent difference in microstructure of the joint area of the pieces deformed at 950 °C and 1150 °C.

### 5.1. Recovery Effect of Fatigue Properties under Different Deformation Modes

**Figure 12:**
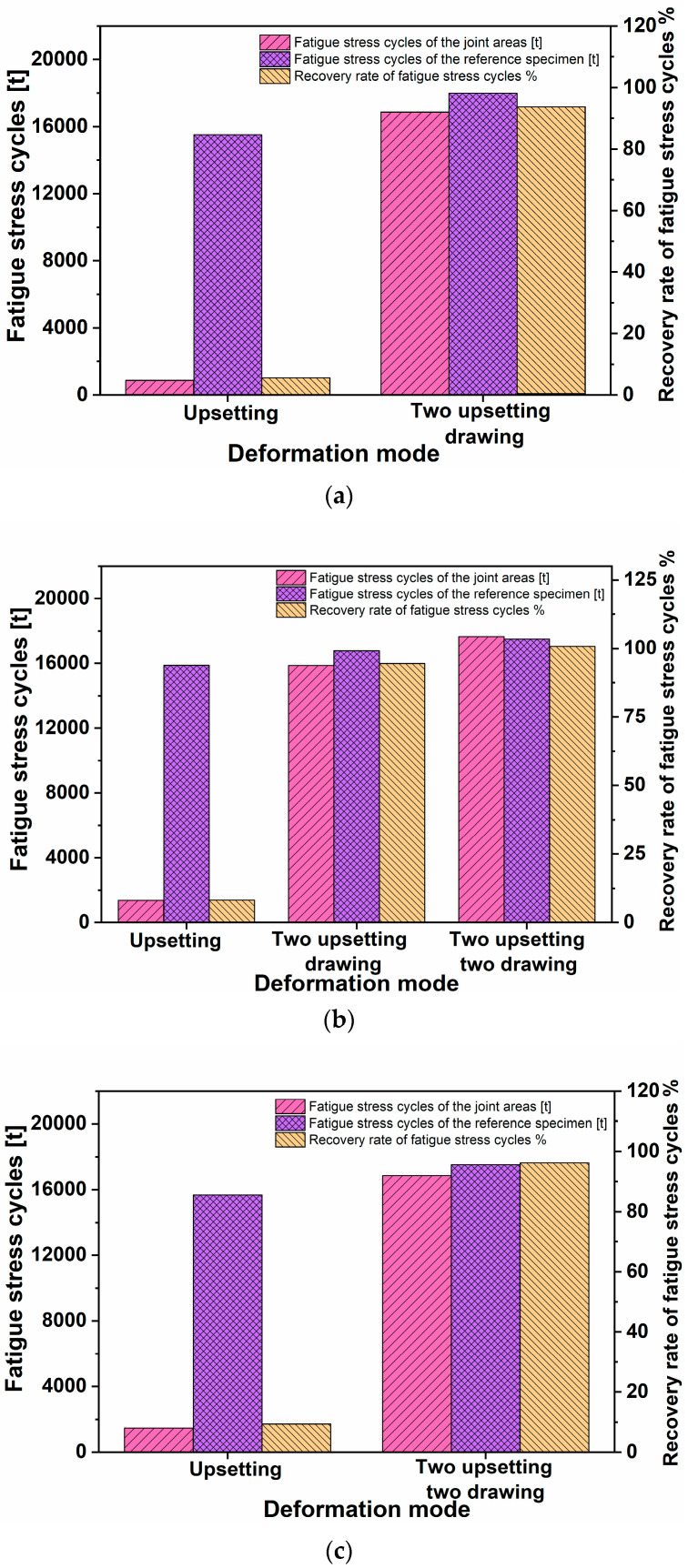
Percentage recoveries of room-temperature fatigue stress cycles under different deformation modes. (**a**) 950 °C, (**b**) 1050 °C, (**c**) 1150 °C.

By using the same conditions as the impact toughness test, the recovery of fatigue stress cycles in the junction area of the section under different deformation modes is shown in Table 3. The percentage recoveries of room-temperature impact properties under different deformation modes are shown in Figure 12.

When the deformation mode is the same, the fatigue performance of the control specimen increases with the increase in deformation temperature. When the deformation temperature is the same, the number of fatigue stress cycles of the comparison specimen after two upsetting and one drawing deformations is significantly higher than that of the first upsetting, and the number of fatigue stress cycles of the simulated defect specimen returns to the initial state at 1150 °C. The number of fatigue cycles of the specimen after two upsetting and two drawing deformations is basically the same as that of the reference specimen, indicating that the fatigue strength of the defect is basically recovered. By comparing with the reported work [18], the number of fatigue cycles is basically the same.

It can be seen that temperature and plastic deformation are important factors that affect the forging of internal porosity defects to improve the fatigue strength. The effect is mainly reflected by the degree of grain structure uniformity, which also explains the difference in recovery mechanisms of static load properties such as tension and dynamic load properties such as impact and fatigue. The main reason is the difference in grain structure uniformity and micro holes. Obviously, it is more difficult to repair the dynamic mechanical properties such as impact and fatigue than the static mechanical properties such as tension and compression.

### 5.2. The Effect of High-Temperature Plastic Deformation on the Fatigue Property

**Figure 13:**
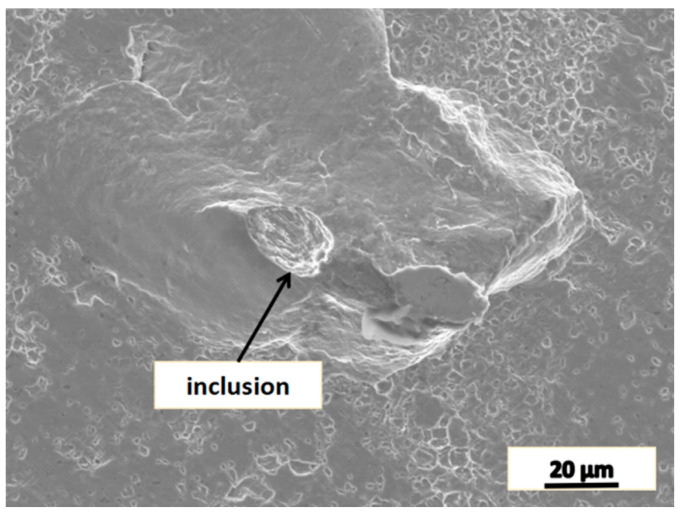
The typical defects in fatigue fracture.

Figure 13 shows the fatigue fracture of the piece heated to 1150 °C, held at the temperature and deformed, and it shows that the size of the inclusion and the unhealed dot is approximately 20 μm. It is much smaller than the equivalent size prescribed in the technical requirement about ultrasonic and other nondestructive testing for heavy forgings. Although the existence of these microscopic defects affects the mechanical properties of the forgings, the technical standards for large forgings have not yet included inspection requirement for these defects [14].

**Figure 14:**
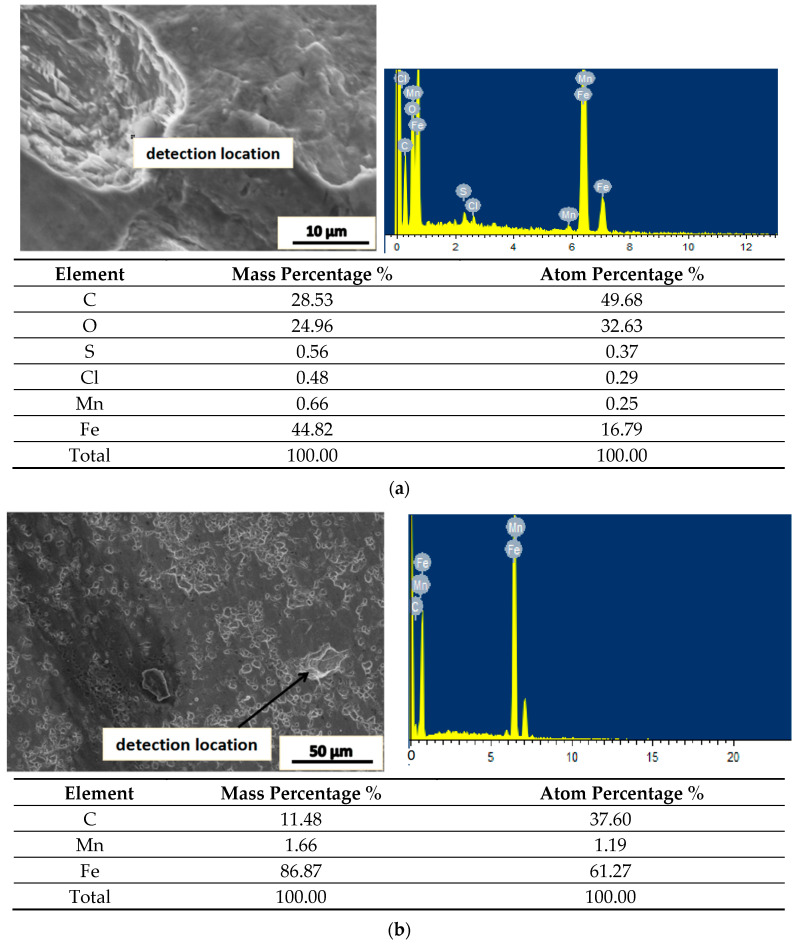
The energy spectrum analysis of the inclusions in fatigue fracture. (**a**) Typical carbonized inclusion and (**b**) the typical inclusions.

Energy spectrum analysis and chemical composition analysis were conducted for particular positions in the fatigue fracture, and the results are shown in Figure 14. The atomic percentage of C at position (a) in Figure 14 is 38.01%, and it is inferred that the inclusions are mainly carbides. The atomic percentages of C, O and Si at the position (b) in Figure 14 are 64.72%, 9.77% and 0.37%, respectively. It is likely to have some inclusions such as CaCO_3_ and SiO_2_. The inclusions significantly decrease the fatigue strength as normally cracks initiate at the boundaries of inclusions and propagate in the cyclic loading process, resulting in fatigue failure. The inclusions at the joint interface and the unhealed micro pores are the major factor leading to low fatigue strength of the specimen after thermoplastic deformation.

The fracture and microstructure analysis demonstrated that the microstructure of the joint area is almost the same as that of the matrix, and the ‘mixed crystal’ phenomenon of the joint area and the matrix is eliminated. The tensile, impact and fatigue properties were fully restored. The fracture presents typical fatigue characteristics. Although the morphology of the inclusions in the fracture was not changed noticeably, its impact on the property under dynamic loads such as fatigue strength and impact toughness is greatly reduced.

## 6. The Influence of the Plastic Deformation on the Different Mechanical Properties

By referring to the data reported in literatures [12,13,14], it is found that under the same deformation temperature, amount and holding conditions, the recovery percentage of the tensile strength is about 100%, the percentage of the impact toughness is about 80%, and that of the fatigue strength is less than 10%. After plastic deformation and holding at a high temperature, the internal porosity defects were almost healed though there are still some microscopic inclusions and holes, and the mechanical property under static load such as tensile strength was basically restored. The recovery percentage of the impact toughness is good; however, the recovery of the fatigue property is poor. It is inferred that the healing of the porosity defects has different impact on the mechanical properties under static and dynamic loads because the microscopic inclusions and pore defects have distinct behaviors under different types of loads. Therefore, the requirements for controlling the internal defects should be determined by referring to the types of the load the forging bears.

Based on the above discussion, it is concluded that the deformation temperature, holding time and the deformation amount are important factors determining the healing effect of internal porosity defects, the evenness of grain structures and morphology of inclusions, thus affecting the mechanical properties of the forgings.

In the process of high-temperature plastic deformation, the healing of the porosity or crack defects can be divided into six stages. The first is the formation and contact of the protrusions on the inner surfaces of the crack. The second is the growth of the protrusions, changing the initial crack into discrete crack segments, followed by the transition of the crack segments into micro hole. In the fourth stage, the micro holes disappears, followed by the fifth stage in which the grain growth happens in the joint area. The final is the stage in which the microstructures in the joint area and the matrix are homogenized. The first to the fourth stages can be achieved only by a single upsetting or drawing, while repeated upsetting and drawing are required if a microstructure adjustment in the last two stages is desired.

The tensile property can be restored after the porosity defects are eliminated in upsetting process, while the recovery of the impact and fatigue properties requires not only the elimination of the porosity defects but also the homogenization of the grain structures in both the joint area and the matrix. The mechanical properties under dynamic loads show recovery only after the healing of porosity defects and the homogenizing of the grain structures, which means that the conditions for recovering the fatigue property are more stringent than those for recovering the tensile property. Although the adverse effects such as inclusions and chemical segregation in the material cannot be completely eliminated by the forging process, porosity defects in the material can be eliminated and microstructure can be homogenized by forging, which reduces the risk of crack propagation greatly. Sufficient plastic deformation in multiple directions under high-temperature conditions with guaranteed time for recrystallization helps to achieve fine grains with desired orientations; however, its technical difficulty undoubtedly increases.

**Figure 15:**
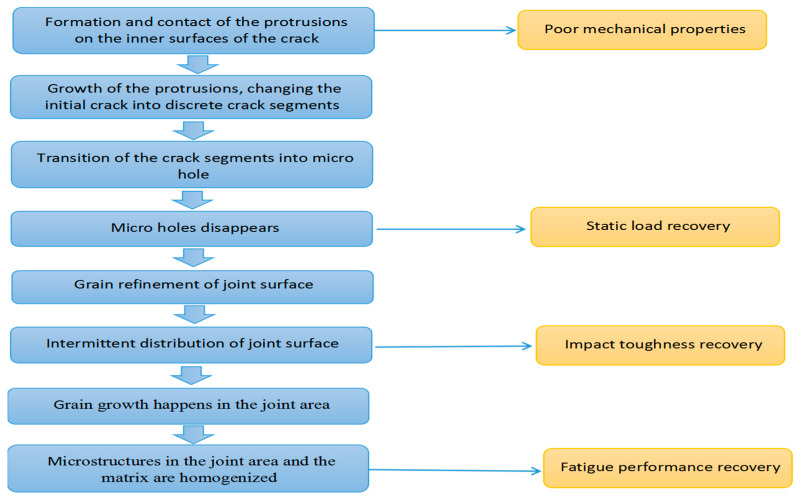
A Schematic showing the correspondence of mechanical property recovery to the structure variation in the healing process.

By studying the influence of plastic deformation process on the tensile strength, impact toughness and fatigue strength, relationship between the recovery of the mechanical properties under static and dynamic loads and the healing of the porosity defects is established, as shown in Figure 15.

**Figure 16:**
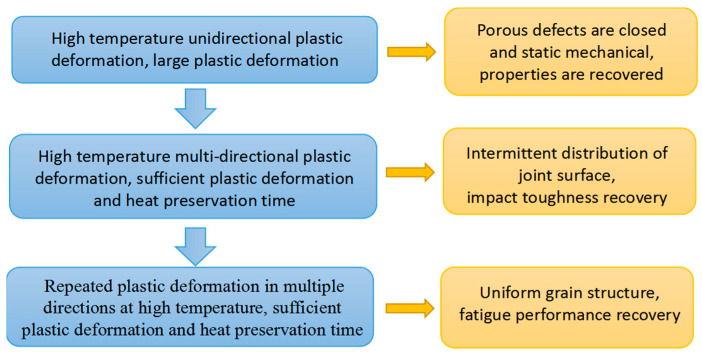
The correspondence of the variation of the internal structures to the hot deformation processes.

Figure 16 shows the correspondence of the variation of the internal structures to the hot deformation processes.

The performance under static loads such as tensile strength and that under dynamic loads such as impact toughness and fatigue strength are a pair of contradictions. Generally, a material with a higher tensile strength has lower impact toughness and fatigue strength. A process window can be determined to achieve a reasonable balance between the static and dynamic properties based on a thorough understanding of the critic microstructure for the property transition.

The mechanical properties under static and dynamic loads must meet certain requirements. The deformation amount must be higher than the critic value at which the flat grain band can be eliminated and a homogenized grain structure becomes possible. Grain structure that is fine and uniform or orderly distributed is the key for the full restoration of the comprehensive mechanical properties of the forgings. The multi-directional plastic deformation achieved by the combined process of upsetting and drawing is the effective way to restore the dynamic mechanical properties.

## 7. Conclusions

- The tensile strength, impact toughness and fatigue strength can be recovered after the porosity defect is healed and the microstructure is made uniform.
- The microstructure feature of tensile property recovery is the disappearance of porosity defects, and the microstructure feature of impact toughness recovery is that the joint surface is not on the same line, and the microstructure feature of fatigue strength recovery is uniform. The deformation conditions for various performance recoveries vary greatly.
- Precipitate becomes the key factor affecting the mechanical properties under dynamic loads once the porosity defect is healed and the microstructure is homogenized.

## Figures and Tables

**Table 1:** Test results of tensile strength of fracture joint zone under different deformation modes.

Temperature [°C]	950			1050			1150		
Upsetting rate (%)	10	20	30	10	20	30	10	20	30
Tensile strength of joint zone [MPa]	320	545	575	370	575	598	340	590	595
Standard deviation of tensile strength [MPa]	25	31	30	27	33	48	29	53	49
Tensile strength of reference specimen [MPa]	610	592	598	636	588	615	585	625	590
Standard deviation of tensile strength of reference specimen [MPa]	46	50	36	56	43	66	36	71	76
Recovery rate of tensile strength %	52.4	92.1	96.2	58.2	97.8	97.2	58.1	94.4	100.8

The tensile strength is the average value of three tests.

**Table 2:** Test results of impact properties of fracture joint zone under different deformation modes.

Temperature [°C]	950			1050			1150		
**Deformation Mode**	**Upsetting**	**Two Upsetting–Drawing**	**Two Upsetting Two Drawing**	**Upsetting**	**Two Upsetting–Drawing**	**Two Upsetting Two Drawing**	**Upsetting**	**Two Upsetting–Drawing**	**Two Upsetting Two Drawing**
Impact toughness of joint area [J]	15.9	5.3	38.6	24.2	15.0	33.8	8.9	19.4	19.2
Standard deviation of impact toughness of joint area [J]	3.6	3.5	5.9	3.9	3.8	6.6	3.2	5.2	4.2
Impact toughness of the reference specimen [J]	37.5	40.1	38.2	27.1	34.8	32.5	14.5	17.9	18.2
Standard deviation of impact toughness of the reference specimen [J]	8.2	6.2	5.3	4.8	7.7	5.4	4.6	4.3	7.2
Recovery rate of impact toughness %	42.4	13.2	98.5	88.3	43.1	104.0	61.4	108.4	105.5

The tensile strength is the average value of three tests.

**Table 3:** Test results of fatigue stress cycles of fracture joint zone under different deformation modes.

Temperature [°C]	950		1050			1150	
**Deformation Mode**	**Upsetting**	**Two Upsetting Two Drawing**	**Upsetting**	**Two Upsetting–Drawing**	**Two Upsetting Two Drawing**	**Upsetting**	**Two Upsetting Two Drawing**
Fatigue stress cycles of the joint areas [t]	876	16,855	1362	15,866	17,655	1476	16,858
Standard deviation of fatigue stress cycles of the joint areas [t]	2.3 × 10^2^	3.2 × 10^3^	5.6 × 10^2^	4.3 × 10^3^	5.1 × 10^3^	7.9 × 10^2^	6.3 × 10^3^
Fatigue stress cycles of the reference specimen [t]	15,511	17,986	15,876	16,783	17,511	15,686	17,522
Standard deviation of fatigue stress cycles of the reference specimen [t]	1.8 × 10^3^	2.9 × 10^3^	4.2 × 10^3^	5.7 × 10^3^	5.4 × 10^3^	4.3 × 10^3^	3.0 × 10^3^
Recovery rate of fatigue stress cycles %	5.6	93.7	8.1	94.5	100.8	9.4	96.2

The fatigue stress cycles is the average value of three tests.

## Data Availability

Not applicable.

## References

[1] Wang B.Z. (2018). Extreme manufacturing of heavy forgings. Forg. Met. Form..

[2] Zhao J.W., Chen X.W., Shi Y.L. (2009). The Current stage and development trend of forging technology and defect control for heavy forging. China Met. Form. Equip. Manuf. Technol..

[3] Kiedssling R. (1978). Non-Metallic Inclusion in Steel.

[4] Fukumoto S., Mitchell A. (1991). The manufacture of alloys with zero oxide inclusion content. Proceedings of the 1991 Vacuum Metallurgy Conference on the Melting and Processing of Specialty Materials.

[5] Li Z.B. (2004). Super clean steels and zero non-metallic inclusion steels. Spec. Steels.

[6] Han J.T., Zhao G., Cao Q.X. (1996). Discovery of inner crack recovery and its structure change in 20MnMo steel. Acta Metall. Sin..

[7] Yu H.L., Liu X.H., Li X.W. (2014). Crack healing in a low-carbon steel under hot plastic deformation, Metall. Mater. Trans..

[8] Wei D.B., Han J.T., Jiang Z.Y. (2006). Tieu, A study on crack healing in 1045 steel. J. Mater. Process. Technol..

[9] Zhang Y.J., Han J.T. (2012). Analysis of microstructure of steel 20 in the range of healing of internal crack. Met. Sci. Heat Treat..

[10] Wei D.B., Jiang Z.Y., Han J.T. (2013). Modelling of the evolution of crack of nanoscale in iron. Comput. Mater. Sci..

[11] Fang Q.H. (2017). Atomic scale investigation of nanocrack evolution in single-crystal and bicrystal metals under compression and shear deformation. J. Alloys Compd..

[12] Xin R.S., Luo J.B., Ma Q.X. (2017). Restoration of impact properties of internal crack healing in a low carbon steel. Mater. Sci. Eng. A.

[13] Qiu Y., Xin R.S., Luo J.B. (2020). Effect of deformation modes and heat treatment on microstructure and impact property restoration of internal crack healing in SA 508 steel. Mater. Sci. Eng. A.

[14] Liu Q.J., Qiu Y., Xin R.S. (2022). Influence of repair effect for internal porosity defects on fatigue performance of heavy forgings. Forg. Stamp. Technol..

[15] (2010). Tensile Test of Metallic Materials Part 1: Room Temperature Test Method.

[16] (2020). Metallic Materials Charpy Impact Test Method.

[17] (2008). Axial Constant Amplitude Low Cycle Fatigue Test Method for Metallic Materials.

[18] Liu Z., Tong Z.F., Liang Z.Q. (2014). Investigation on low-cycle fatigue property of domestic A508-3 steel. At. Energy Sci. Technol..

